# Cyclosporine A Impairs the Macrophage Reverse Cholesterol Transport in Mice by Reducing Sterol Fecal Excretion

**DOI:** 10.1371/journal.pone.0071572

**Published:** 2013-08-09

**Authors:** Ilaria Zanotti, Daniela Greco, Giulia Lusardi, Francesca Zimetti, Francesco Potì, Lorenzo Arnaboldi, Alberto Corsini, Franco Bernini

**Affiliations:** 1 Dipartimento di Farmacia, Università degli Studi di Parma, Parma, Italy; 2 Dipartimento di Scienze Biomediche, Metaboliche e Neuroscienze, Università di Modena e Reggio Emilia, Modena, Italy; 3 Dipartimento di Scienze Farmacologiche e Biomolecolari, Università degli Studi di Milano, Milano, Italy; National Institutes of Health, United States of America

## Abstract

Despite the efficacy in reducing acute rejection events in organ transplanted subjects, long term therapy with cyclosporine A is associated with increased atherosclerotic cardiovascular morbidity. We studied whether this drug affects the antiatherogenic process of the reverse cholesterol transport from macrophages in vivo. Cyclosporine A 50 mg/kg/d was administered to C57BL/6 mice by subcutaneous injection for 14 days. Macrophage reverse cholesterol transport was assessed by following [^3^H]-cholesterol mobilization from pre-labeled intraperitoneally injected macrophages, expressing or not apolipoprotein E, to plasma, liver and feces. The pharmacological treatment significantly reduced the amount of radioactive sterols in the feces, independently on the expression of apolipoprotein E in the macrophages injected into recipient mice and in absence of changes of plasma levels of high density lipoprotein-cholesterol. Gene expression analysis revealed that cyclosporine A inhibited the hepatic levels of cholesterol 7-alpha-hydroxylase, concomitantly with the increase in hepatic and intestinal expression of ATP Binding Cassette G5. However, the in vivo relevance of the last observation was challenged by the demonstration that mice treated or not with cyclosporine A showed the same levels of circulating beta-sitosterol. These results indicate that treatment of mice with cyclosporine A impaired the macrophage reverse cholesterol transport by reducing fecal sterol excretion, possibly through the inhibition of cholesterol 7-alpha-hydroxylase expression. The current observation may provide a potential mechanism for the high incidence of atherosclerotic coronary artery disease following the immunosuppressant therapy in organ transplanted recipients.

## Introduction

Until the 1970s the high incidence of allograft loss as a result of acute rejection represented a major concern for organ transplanted patients. Since those years, the advances in immunosuppressive therapy made this procedure safe and efficient, and moved the outcome measures to long-term survival and morbidity. Death with a functioning graft due to cardiovascular disease is currently the leading cause of mortality in solid organ recipients [1]. Cyclosporine A (CsA) was the mainstay of immunosuppression throughout the 1980s and early 1990s and is currently successfully used in combination therapy in renal and liver transplantation [2]. Despite its therapeutic efficacy, CsA chronic use is associated with well documented independent risk factors for atherosclerosis, such as hypertension, diabetes and dyslipidemia [1].

The reverse cholesterol transport (RCT) is the process that may counteract the pathogenic events leading to the formation of atheroma. The promotion of cholesterol removal from peripheral tissues occurs in 3 main steps: 1) cholesterol efflux: the rate limiting step, consisting in the release of excess cholesterol from peripheral cells; this process is driven by both cell capacity to remove cholesterol and plasma capacity to act as lipid acceptor; 2) high density lipoproteins (HDL) remodeling: occurring through several reactions catalyzed by enzymes that induce structural modifications of HDL; 3) hepatic uptake: cholesterol is delivered by HDL to the liver, where is partially converted into bile acids for the ultimate excretion into the bile [3]. Based on macrophage primary role in atherosclerotic lesion formation, macrophage-derived cholesterol pool is considered the most important for atherosclerosis development and progression. Thus, the RCT that specifically involves macrophage-derived cholesterol becomes fundamental for atheroprotection. This process is termed macrophage RCT [4] and is currently estimated in vivo with a radioisotope-based assay. Several works established that macrophage RCT inversely correlates with atherosclerosis in animal models (studies summarized in Rader’s review [5]), and identified this process as a significant predictor of cardiovascular disease.

The aim of this work was to investigate whether CsA may exert its well documented proatherosclerotic activity by affecting macrophage RCT. To this purpose, we measured the process in C57BL/6 mice, an animal model where CsA was previously shown to accelerate atherosclerosis development [6].

We provided the demonstration that CsA impairs the antiatherosclerotic process of macrophage RCT in vivo by reducing fecal sterol excretion through the inhibition of cholesterol 7-alpha-hydroxylase (*Cyp7α*) expression in the liver.

## Methods

### Materials and Cells

CsA was purchased from Sigma-Aldrich (Milano, Italy). Organic solvents were purchased from Romil Ltd (Cambridge, UK), Carlo Erba (Milano, Italy) and Merck (Darmstadt, Germany). [^3^H]-cholesterol was from Perkin Elmer (Monza, Italy). Tissue culture flasks and plates were from Corning (Corning, NY, USA), transwell inserts from Greiner Bio-one (Frickenhausen, Germany). Cell culture medium, fetal bovine serum and PBS were from Lonza Ltd (Milano, Italy). Bovine serum albumin and Brewer thioglycollate medium were purchased from Sigma-Aldrich (Milano, Italy). T0901317 was from Alexis Biochemicals (Lausen, Switzerland). Acetylated low density lipoproteins (AcLDL) were prepared from human LDL by reaction with acetic anhydride, as previously described [7].

J774 mouse macrophages, a cell type widely used in studies on lipid metabolism [8], were kindly donated by Prof. George Rothblat (Children’s Hospital of Philadelphia).

### Ethic Statements on the use of Animals

Animal care and experimental procedures were performed with the approval of the local Comitato Etico per la Sperimentazione Animale, overseeing animal experiments at University of Parma. No special permission for use of animals (mice) in such pharmacological studies is required in Italy, as defined by the legislative decree 116/92.

### Drug Administration to Mice

Twelve week old male C57BL/6J mice were housed in a controlled environment at 25±2°C with alternating 12 h light and dark cycles and received standard diet and water ad libitum. Since CsA deleterious effects on cardiovascular risk factors in animal models have been previously described both upon short term (few days) and longer time periods (until 300 days) of administration, at doses ranging from 20 mg/kg/d to 50 mg/kg/d [6], mice were treated for 14 days by subcutaneous injection with CsA dissolved in olive oil at the dose of 50 mg/kg (n = 5–7) or vehicle (n = 5–7), once a day at 10 a.m. On day 14, 4****h after the last drug administration, mice were sacrificed by excess anesthesia with ethyl ether. Blood was collected by cardiac puncture and recovered in plastic tubes containing sodium citrate 3.8%. Plasma was isolated by low speed centrifugation and stored at −80°C until use, as described below. Livers were collected at the end of the treatment period and immediately frozen in liquid nitrogen. The bile was collected from the gall bladder and 5 µl of it was subjected to liquid scintillation counting. Feces were collected on day 14 of drug treatment. Samples of liver were extracted by the Bligh and Dyer method [9], while aliquots of feces were extracted by a method that allows to separate bile acid and neutral sterol fractions [10]; radioactivity in the lipid extracts was measured by liquid scintillation counting.

### Evaluation of RCT *in vivo*

Measurement of RCT was performed as previously described [11]. On day 11 of pharmacological treatment with CsA, thioglycollate-elicited murine peritoneal (MPM) or J774 macrophages were cholesterol-enriched with 25 µg/mL AcLDL and radiolabeled with 5 µCi/ml [^3^H]-cholesterol. On day 13, cells were intraperitoneally injected into recipient mice (n = 14 in RCT using MPM; n = 10 in RCT using J774). On day 14, mice were sacrificed and samples were collected as described above.

### Measurements of Plasma Lipids

Plasma total cholesterol, HDL- cholesterol and triglycerides were measured with colorimetric assays, using commercially available kits (Instrumentation Laboratory, Werfen Group, Milan, Italy).

### Quantification of Beta-sitosterol in Plasma

120 µl of thawed plasma samples were hydrolyzed with 96% ethanol-KOH 50% for 2 hours at 85°C in glass vials and periodically stirred, after addition of 5 µg of 5-alpha cholestane as internal standard. At the end of the reaction, ethanol, KOH 1 M and petroleum ether (40/60°C) were added and samples vigorously shaken and heated for 20 minutes. The organic phase were then removed and the procedure repeated three times. The extracts were dried in a flow of nitrogen and resuspended in hexane, before being analyzed by a DANI 1000 gas-liquid chromatographer (DANI Instruments, Milan, Italy) equipped with a flame ionization detector and a 30 m, 0.32 mm, 0.25 m MEGA-1 (Mega Columns, Legnano, Italy) fused silica column. The flow of hydrogen was at a constant pressure of 1 bar, and the detector temperature was 350°C. Oven temperature ranged from 240 to 300°C (total run 15 min). Chromatograms were recorded and the amount of beta-sitosterol quantified by Clarity Software (Clarity, Prague, Czech Republic), comparing the area of the beta- sitosterol peak with that of 5 alpha-cholestane and correcting the obtained value by 1.112, corresponding to the differences in weight between the two sterols.

### Cholesterol Efflux from Macrophages

After plating, murine macrophages J774 or MPM were labeled for 48 h with 2 µCi/ml [^3^H]-cholesterol in medium in the presence of 1% fetal calf serum. Cells were loaded with cholesterol during the labeling period by the addition of 25 µg/ml AcLDL to the medium. Cell monolayers were then equilibrated for 6 h in an albumin containing medium. Cholesterol efflux was promoted by incubation with pooled C57BL/6 mouse plasma diluted to 0.1–0.5–1% in presence or absence of CsA 1–5 µM for 24 h. An Acyl-coenzyme A: cholesterol O-Acyltransferase inhibitor (2 µg/ml, Sandoz 58035) was added during labeling and equilibration period to prevent cellular accumulation of cholesteryl ester [8]. The drug concentration was selected according to previous studies assessing CsA effect in cultured cells [12], [13], [14]. In our experience, 5 µM was the highest concentration not affecting cell viability. Cholesterol efflux has been calculated as a percentage of the radioactivity released to the medium over the radioactivity incorporated by cells before addition of plasma (Time zero). Every sample was analyzed in triplicate and the average and standard deviation have been obtained. Background efflux, evaluated in the absence of acceptors, was subtracted from each sample value.

### Plasma Cholesterol Efflux Capacity

Plasma samples from mice treated with CsA or vehicle as described previously, were harvested and stored at −80°C; the aliquots were slowly defrosted in ice just before addition to cells. To assess plasma efflux capacity, MPM were radiolabeled with cholesterol and cholesterol enriched by incubation with 25 µg/ml of AcLDL in 1% fetal calf serum containing medium for 24 h. After a 18 h equilibration period in an albumin-containing medium, cholesterol efflux was promoted to 0.5% (v/v) plasma from either vehicle and CsA-treated mice for 5 h. Plasma cholesterol efflux capacity has been calculated as a percentage of the radioactivity released to the medium in 5 h over the radioactivity incorporated by cells before addition of plasma (Time zero). Every plasma sample was analyzed in triplicate and the average and standard deviation have been obtained. A 0.5% of a serum pool from normolipidemic mice was run to confirm the correct responsiveness of cells; background efflux, evaluated in the absence of acceptors, was subtracted from each sample value.

### Analysis of Gene Expression by Real-time Quantitative RT-PCR

Total RNA from liver samples was isolated by the RNeasy Mini kit (Qiagen) according to the protocol provided by the manufacturer. Briefly, liver samples were lysed and homogenized by Ultraturrax and then loaded onto the RNeasy silica membrane spin column. RNA was then eluted in water and quantified by spectrophotometry (Nanodrop ND-1000, Thermo Scientific). cDNA was synthesized from 100 ng of total RNA using iScript™ cDNA Synthesis Kit (Bio-Rad Laboratories). mRNA levels were quantitatively determined on an CFX96™ Real-Time PCR Detection System using SsoFast™ EvaGreen® Supermix according to manufacturer’s instructions (Bio-Rad Laboratories). PCR primers were the same published in Ye et al. [15] (ATP Binding Cassette G5 (*Abcg5*): forward primer 5′–TGGCCCTGCTCAGCATCT–3′; reverse primer 5′–ATTTTTAAAGGAATGGGCATCTCTT–3′; ATP Binding Cassette G8 (*Abcg8*): forward primer 5′–CCGTCGTCAGATTTCCAATGA–3′; reverse primer 5′–GGCTTCCGACCCATGAATG–3′; *Hprt*: forward primer 5′–TTGCTCGAGATGTCATGAAGGA–3′; reverse primer 5′–AGCAGGTCAGCAAAGAACTTATAG–3′; β-actin: forward primer 5′–AACCGTGAAAAGATGACCCAGAT–3′; reverse primer 5′–CACAGCCTGGATGGCTACGTA–3′). Primer sequences for *Cyp7α* e Niemann-Pick C1 Like-1 (*Npc1l1*) were specifically designed with Beacon Designer Software (*Cyp7α*: forward primer 5′–TAGATAGCATCATCAAGGA–3′; reverse primer 5′–AAGGTGTAGAGTGAAGTC–3′; *Npc1l1*: forward primer 5′–TAGCAGCCAACATCACAG–3′; reverse primer 5′–ATCGTGTAAGGGAAGACC–3′). Relative gene expression numbers were calculated by applying the 2^−ΔΔCt^ method [16]. Briefly, the threshold cycle number (Ct) of the target gene was subtracted from the average Ct of *Hprt* and β-actin (Ct _housekeeping_) and raised 2 to the power of this difference. The average (geometric mean) of two housekeeping genes was used to exclude the possibility that changes in relative expression were caused by variations in the expression of separate housekeeping genes.

### Western Blotting

Liver samples from mice treated with CsA or vehicle as described above were lysed in RIPA buffer containing aprotinin 10 µg/ml, leupeptin 1 µg/ml, pepstatin 1 µg/ml, phenylmethanesulfonyl fluoride 0.2 mM and homogenized by Ultraturrax. Equal amounts of protein (50 µg) were separated on 10% acrylamide gels and transferred to nitrocellulose membranes. ABCG5 and ABCG8 were detected with rabbit primary antibodies (Santa Cruz, Santa Cruz, California) and a secondary antibody, anti-rabbit IgG conjugated to horseradish peroxidase, with visualization by enhanced chemioluminescence (ECL Plus) (both from GE Healthcare, Little Chalfont, Buckinghamshire, UK), according to the manufacturer’s conditions.

### Statistical Analysis

The statistical analyses were performed with Prism 5 software. (GraphPad Software, San Diego, California). Experimental data sets were tested for normality by D’Agostino Pearson’s test. Comparisons between two groups were done with Mann Whitney test or unpaired two-tailed Student t-test. Comparisons between more than two groups were made by one-way or two-way ANOVA for independent samples. Pairwise comparisons of sample means were performed with Bonferroni post-hoc test. A level of *p*<0.05 was considered significant.

## Results

### Evaluation of CsA Effect on Plasma Lipids and Macrophage RCT *in vivo*

**Figure 1:**
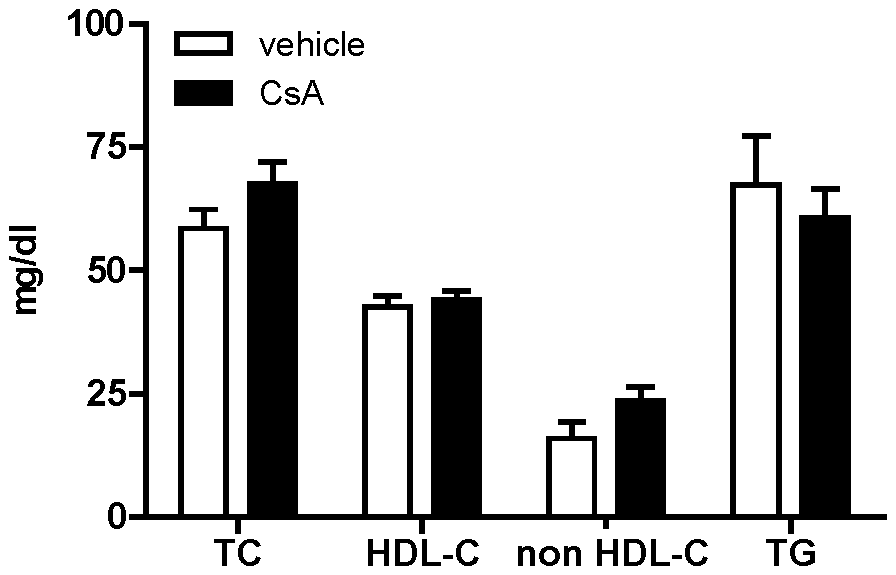
CsA treatment effect on plasma lipid levels in mice. C57BL/6 mice were treated with CsA 50 mg/kg/d (black bar) or vehicle (white bar) for 14 days. Total cholesterol (TC), HDL-cholesterol (HDL-C) and triglycerides (TG) were measured by an enzymatic assay. Non HDL- cholesterol (non HDL-C) is calculated as the difference between TC and HDL-C. Data are expressed as mean ± SD (values are mean of 7 animals).

**Figure 2:**
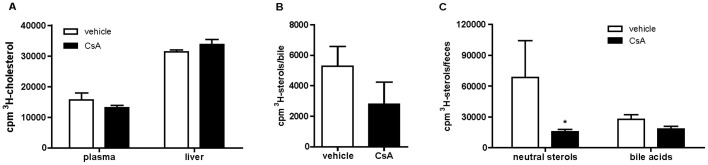
CsA treatment effect on macrophage RCT from MPM in vivo. C57BL/6 mice were treated with CsA as described in Figure 1. On day 13 of pharmacological treatment, animals were intraperitoneally injected with [^3^H]-cholesterol loaded MPM. After 24 h, mice were sacrificed and macrophage-derived [^3^H]-cholesterol distribution was quantified in plasma, liver and feces as described in the Methods section. A: [^3^H]-cholesterol in plasma and liver; B: [^3^H]-sterols in the bile; C: [^3^H]-neutral sterols and [^3^H]-bile acids in the feces. Results are expressed as mean ± SD (n = 7 mice per group). *p<0.05 vs. vehicle.

In order to investigate whether CsA proatherosclerotic effect could be associated with the impairment of macrophage RCT, we treated C57BL/6 mice with CsA 50 mg/kg/d for 14 days. During this period, mice displayed normal food intake and no significant changes in body or liver weight compared to vehicle-treated animals (Table S1 and S2). CsA did not affect plasma lipid levels: whereas HDL-cholesterol and triglyceride levels were unchanged, a small, not significant trend for increased total cholesterol was observed, attributable to increased non HDL-cholesterol (Figure 1). The pharmacological treatment did not influence the radioactive cholesterol appearance in plasma or liver **(** Figure 2A **)**, but caused a reduction of [^3^H]-sterol excretion into the bile (Figure 2B) and the feces, both as bile acids and neutral sterols (Figure 2C).

### Evaluation of CsA Effect on Macrophage RCT *in vivo* from J774

**Figure 3:**
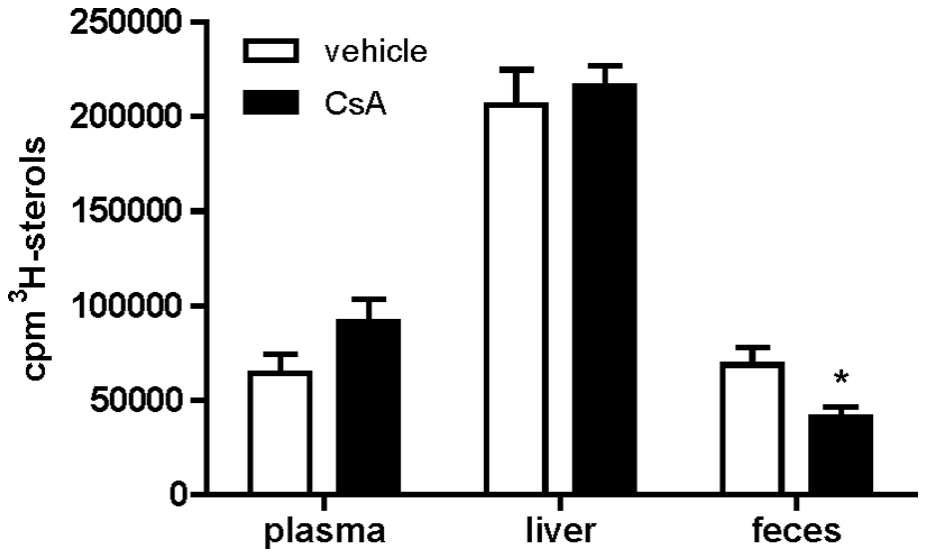
CsA effect on macrophage RCT from J774. C57BL/6 mice were treated with CsA and RCT was measured as described in Figure 1, upon injection with [^3^H]-cholesterol loaded J774 foam cells. Results are expressed as mean ± SD (n = 5 mice per group). *p<0.05 vs. vehicle.

To evaluate whether CsA-mediated block of apolipoprotein (apoE) secretion from macrophages may account for the observed impairment of RCT in vivo, the drug effect was evaluated by measuring the process in mice receiving [^3^H]-cholesterol-loaded J774, a type of macrophages not expressing apoE [17]. In this experimental conditions, we confirmed that CsA did not affect neither body, nor liver weight (Table S3 and S4). Moreover, plasma lipid levels was not significantly affected, even if a trend for increased total and non-HDL cholesterol was still observed (Figure S1). More importantly, CsA inhibited radioactive cholesterol excretion similarly to the previous experiment using MPM (Figure 3).

### Evaluation of CsA Effect on Cell Cholesterol Efflux from Macrophages

**Figure 4:**
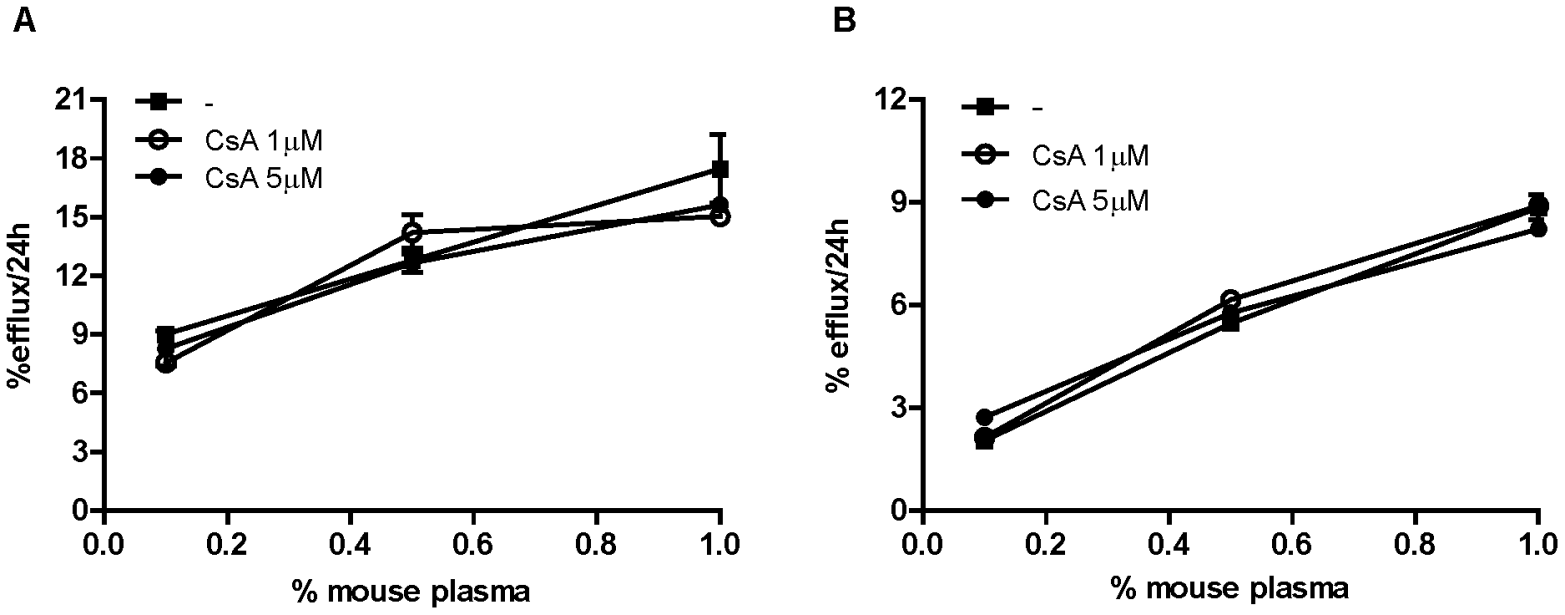
CsA effect on cholesterol efflux from macrophages. A: MPM and B: J774 were cholesterol enriched with AcLDL 25 µg/ml and radiolabeled with [^3^H]-cholesterol 2 µCi/ml for 48 hours. After an equilibration period of 6 hours, cells were exposed to increasing concentrations of plasma from wild type mice in presence or absence of CsA 1–5 µM for 24 hours. Efflux is expressed as cpm in medium/cpm time zero x100 (mean of triplicates ± SD).

To investigate the mechanism by which CsA caused the reduction of radioactive accumulation in the feces, we assessed the drug effect on the single steps of the RCT process. First, we focused on cholesterol efflux from macrophages, the first, rate-limiting step of the process. An in vitro assay was carried out, using the following experimental conditions: cholesterol-loaded MPM or J774, the same cell types injected in the peritoneum of recipient mice for RCT measurement, were incubated with CsA in presence of murine plasma as cholesterol acceptor for 24 hours. The addition of two different concentrations of CsA did not produce alterations of cholesterol efflux compared to cells receiving no drug neither in MPM (Figure 4A), nor in J774 (Figure 4B).

**Figure 5:**
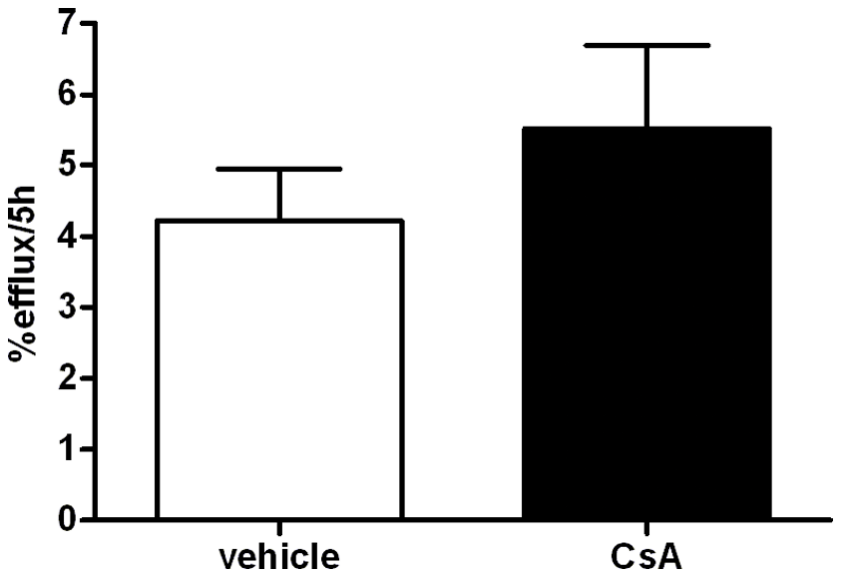
CsA treatment effect on plasma cholesterol efflux capacity. MPM were cholesterol enriched with 25 µg/ml AcLDL and radiolabeled with [^3^H]-cholesterol 2 µCi/ml. After an equilibration period of 18 hours, cells were exposed for 5 hours to 0.5% plasma from mice treated with CsA or vehicle as described in Figure 1. Efflux is expressed as cpm in medium/cpm of time zero x100 (values are mean of 7 animals per group ± SD).

Next, we hypothesized that CsA administration to mice may have promoted lipoprotein remodeling, leading to modifications of their capacity to promote cholesterol efflux. To test this hypothesis, plasma from vehicle or CsA-treated mice was used as lipid acceptor in cholesterol efflux assays from cholesterol-loaded macrophages, the same cell type injected in the peritoneum of recipient mice for RCT measurement. As shown in Figure 5, plasma from vehicle and CsA-treated mice showed similar capacity to promote cholesterol efflux from foam cells.

### Evaluation of CsA Effect on ABCG5/ABCG8 Expression and Function

**Figure 6:**
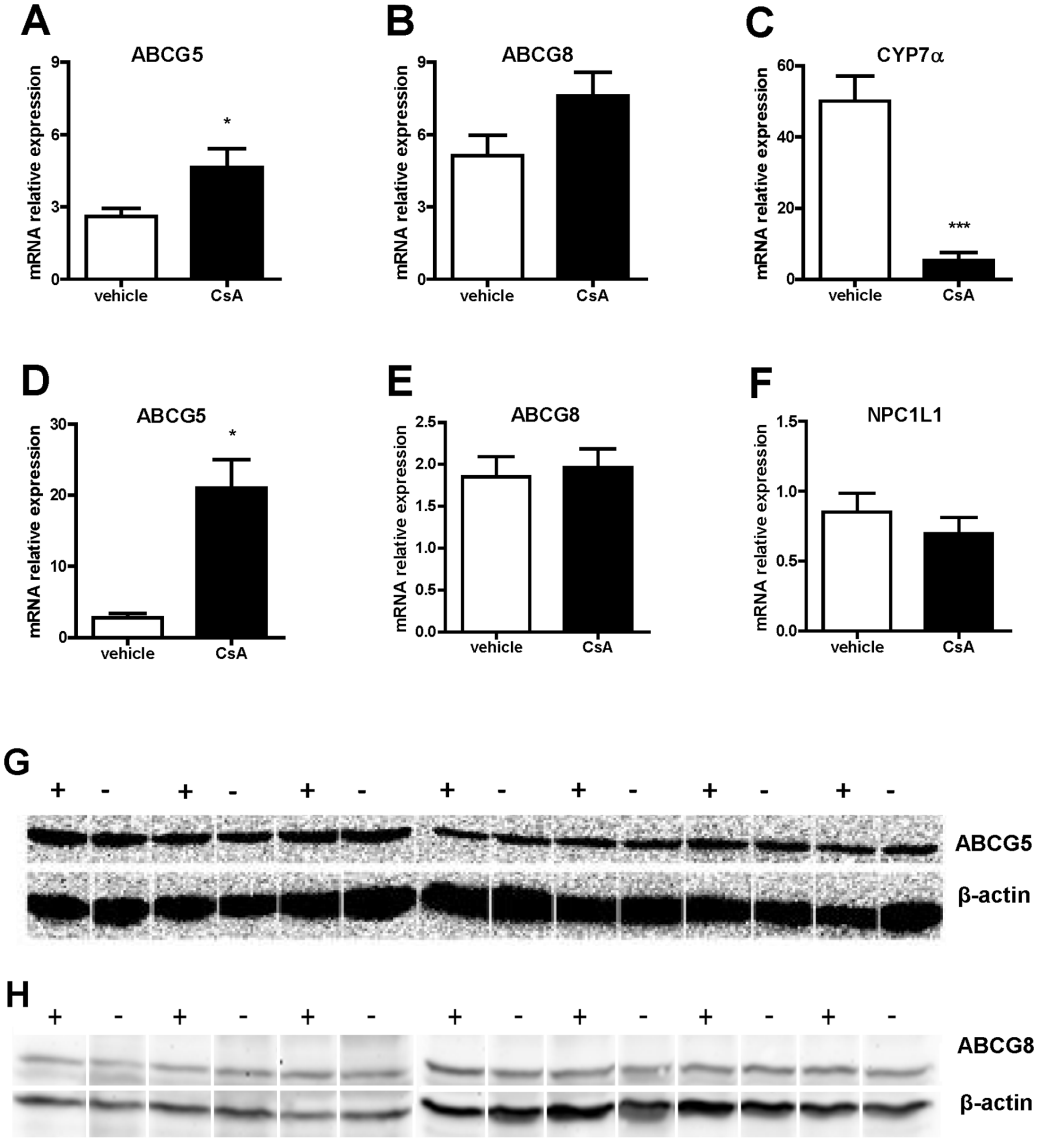
CsA treatment effect on sterol transporters expression in vivo. C57BL/6 mice were treated with CsA as described in Methods. After the sacrifice, liver and small intestine were collected to isolate RNA. *Abcg5*, *Abcg8*, *Cyp7α* and *Npc1l1* mRNA was quantified by qRT-PCR in liver (A,B,C) and intestine (D,E,F) respectively. Results were normalized against *Hprt* (shown); normalization against β-actin, or the average of them gave similar results (not shown). G, H: Western blotting analysis was performed on hepatic lysates prepared as described in the Methods section. Lanes (–): mice treated with vehicle. Lanes (+): mice treated with CsA. Data are presented as means ± SD. *p<0.05 and ***p<0.001 vs vehicle.

Next, we investigated whether the drug may impair the sterol fecal excretion by affecting ABCG5/ABCG8 expression and/or activity. These proteins play a pivotal role in cholesterol elimination from the body, being responsible for neutral sterol excretion into the bile and the intestinal lumen [18]. RT-PCR analysis on hepatic expression of both transporters revealed that CsA treatment in vivo produced a slight, but significant increase in *Abcg5* mRNA expression, whereas the increase in *Abcg8* mRNA did not reach the statistical significance (p = 0.0745) (Figure 6A and 6B). Differently, the protein content of both transporters did not change (Figure 6G and 6H). The same trend was observed when we evaluated CsA impact on *Abc*g5 and *Abcg8* expression in the intestine: surprisingly the drug produced a significant raise of the *Abcg5* mRNA, whereas no effect were observed in *Abcg8* mRNA (Figure 6D and 6E ). CsA effect on ABCG5/ABCG8 activity was first evaluated in an in vitro system, using Caco-2 cells and assessing the drug capacity to affect cell cholesterol efflux. Following Liver X Receptor stimulation, these cells express the ABCG5/ABCG8 heterodimer on the apical membrane, resulting in cholesterol secretion into the apical medium [19]. Consistently, we observed a significant increase in cholesterol efflux to either cell medium or taurocholate-containing micelles upon addition of the synthetic Liver X Receptor agonist, T0901317 (Figures S2A and S2B). In absence of cholesterol acceptor, CsA incubated in the efflux, but not in the equilibration period, significantly reduced cholesterol release (Figure S2A). Interestingly, the drug reduced cholesterol efflux even in the absence of Liver X Receptor stimulation, despite less profoundly (Figure S2A and S2B). A clear trend for reduced cholesterol efflux was observed also in cells treated with CsA and exposed to micelles, although a statistical significance was not reached.

**Figure 7:**
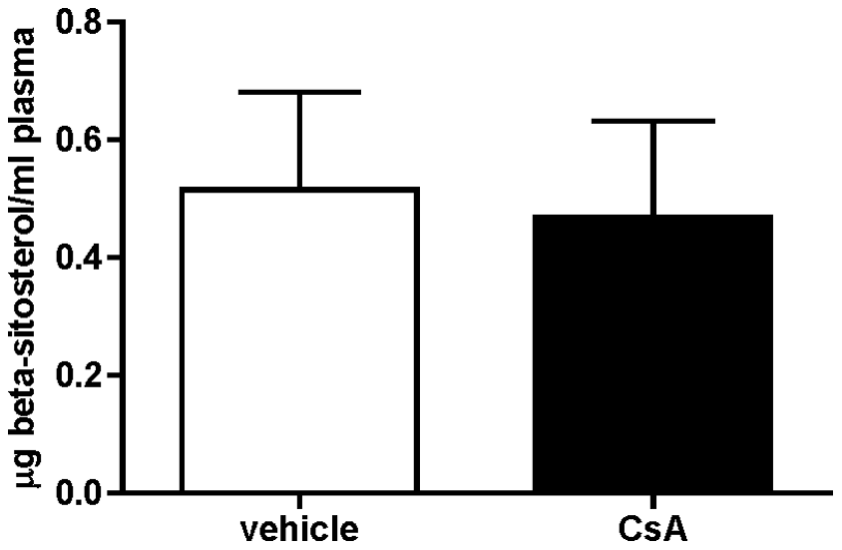
CsA treatment effect on plasma levels of beta-sitosterol. C57BL/6 mice were treated with CsA as described in Figure 1. The plasma content of beta-sitosterol was measured by gas chromatography, as described in the Method section. Data are presented as means ± SD (n = 6).

Taken together these results suggest that CsA may affect ABCG5/ABCG8 activity. The in vivo relevance of this observation was assessed by measuring circulating beta-sitosterol in plasma of mice treated or not with the drug. In fact, functional impairment of these transporters in vivo may result in accumulation of phytosterols in plasma [20]. However, no differences were detected in the two groups (Figure 7).

### Evaluation of CsA Effect on *Cyp7α* and *Npc1l1* Expression

To investigate whether the observed reduction of radioactive cholesterol fecal excretion in CsA-treated mice could be attributed to the drug influence on cholesterol conversion into bile acids and/or promotion of cholesterol intestinal absorption, hepatic and intestinal expression of *Cyp7α* and *Npc1l1* respectively was evaluated. As reported in Figure 6C and 6F, CsA significantly reduced *Cyp7α*, but not *Npc1l1* mRNA.

## Discussion

The increased cardiovascular morbidity and mortality following CsA treatment represents a serious concern for long-term therapy of organ transplanted patients [1]. Given the well documented role of macrophage RCT in the protection of atherosclerosis [5], we hypothesized that CsA may impair this process. Indeed, a short term administration of CsA to mice resulted in reduced elimination of macrophage-derived cholesterol from the body. In particular, we reported the inhibition of sterol accumulation in the bile and feces, suggesting the drug interference with the last step of RCT, cholesterol excretion. This effect could be the result of multiple mechanisms, including the modulation of the activity of proteins involved in bile acid synthesis, such as CYP7α and cholesterol 27-alpha-hydroxylase, or proteins responsible for cholesterol intestinal absorption and excretion, such as NPC1L1 and ABCG5-ABCG8 respectively. Our initial hypothesis was that CsA could affect the activity of ABCG5-ABCG8, the half transporters expressed on the apical membrane of hepatic and intestinal cells, responsible for the excretion of neutral sterols into the bile and intestinal lumen [21]. The in vitro evaluation of CsA effect on ABCG5/ABCG8 activity was likely to support our hypothesis, since the drug impaired sterol efflux from cultured intestinal cells. However, when we assessed the relevance of this result *in vivo*, we failed to definitely confirm the involvement of these genes. First of all, gene/protein expression analysis on the livers and intestines of vehicle and CsA-treated mice revealed that the pharmacological treatment did not result in the reduction of gene/protein expression, but rather in the increase or no changes. Moreover, mice treated with CsA showed the same amount of beta-sitosterol of untreated animals, suggesting that ABCG5/ABCG8 activity was not affected in vivo. The inhibition of ABCG5/ABCG8 is expected to cause alterations in the balance of cholesterol and phytosterols, resulting in dramatic increase of circulating levels of the latter. This is the case of subjects affected by sitosterolemia [20] or mice with deletions of *Abcg5/Abcg8* genes [22]. Taken together these evidences rule out the involvement of ABCG5/ABCG8 in CsA-mediated impairment of macrophage RCT.

Successively, CsA-mediated modulation of intestinal cholesterol absorption via NPC1L1 was considered. In this case, the reduced fecal excretion could be attributed to improved activity of this protein, leading to augmented availability of cholesterol in the intestinal lumen for the final elimination. However, gene expression analysis clearly revealed no modulation of *Npc1l1* mRNA in CsA-treated mice, thus ruling out this potential mechanism of action. Finally, we evaluated whether the observed reduction of radioactivity fecal excretion could be related to inhibition of *Cyp7*α expression, the enzyme driving the rate limiting step of cholesterol conversion into bile acids. The observed, significant reduction of hepatic expression upon the pharmacological treatment with CsA is consistent with previous reports [23] and apparently suggest that the drug may impair macrophage RCT through the inhibition of this key enzyme in cholesterol catabolism. Although the impairment of CYP7Α is sufficient to explain the reduced neutral sterol excretion in the bile and intestine, we can not exclude that CsA may affect other mechanisms accounting for this effect, including the inhibition of cholesterol 27-alpha-hydroxylase activity [24] or the interference with trans intestinal cholesterol efflux [25]. Further studies will be necessary to support these hypothesis and are beyond the scope of the present work.

A possible interference of CsA on cholesterol efflux, the first step of RCT, was also investigated. CsA influence on this process could lead to a defective mobilization of cholesterol from cells either by impairing cell capacity to promote cholesterol release or reducing plasma ability to accept cellular cholesterol. In vivo treatment with CsA was shown to inhibit apolipoprotein A-I expression [12], and to reduce HDL levels [14], two effects that are expected to negatively impact macrophage RCT efficiency by producing a proatherogenic lipoprotein profile. However, in our experimental setting, CsA treatment did not produce significant alterations in plasma lipid levels: HDL-cholesterol levels were unchanged and only a small trend to increased non HDL-cholesterol was observed. Whereas its impact on RCT extent can not be fully ruled out, we believe that it can not account for relevant effect. In previous works, an increase in non HDL-cholesterol was associated to modulation of RCT only when accompanied by increase in HDL-cholesterol [26], [27]. The lack of apparent dyslipidemia upon CsA treatment is not surprising, since the administration of this drug in animals is not always associated with increase in plasma lipid levels, even in presence of proatherosclerotic effects [6], [12]. It is also noteworthy that the effects of pharmacological interventions on macrophage RCT are often independent on variations of lipoprotein profile [5]. Importantly, the capacity of plasma from vehicle- and CsA-treated mice to promote cholesterol release from foam cells was similar, ruling out the drug impact on both HDL structure and function. CsA effect on cell capacity to promote cholesterol efflux could be related to the drug interference with ATP Binding Cassette A1 or apoE. The impact of CsA on ATP Binding Cassette A1, a lipid transporter associated with atheroprotective activity in relation to the role in HDL biogenesis [28], in the promotion of cholesterol efflux from macrophages [29] and in macrophage RCT [30], was recently reported [14]. However, our results indicate that CsA effect on macrophage RCT in our experimental conditions does not involve this protein. In fact, when we tested CsA effect in cultured cholesterol-enriched macrophage foam cells, that express significant levels of ATP Binding Cassette A1 [31], the drug did not show to affect cholesterol efflux. In this experiment we choose to treat cells with CsA for a long incubation time in order to better reproduce the in vivo RCT experiment, in which cells are continuously exposed to either CsA and plasma. Finally, the lack of CsA impact on HDL plasma levels definitely rules out the drug interference with ATP Binding Cassette A1 in vivo. Recently, we demonstrated that apoE expressed in macrophages is essential for functional macrophage RCT efficiency in vivo by promoting cell cholesterol efflux [32], whereas Kritharides’s group demonstrated that CsA reduced apoE secretion from cultured macrophages [13]. However, we could rule out that the observed inhibition of RCT is related to CsA-mediated reduction of apoE secretion from macrophages. In fact, the drug did not influence cholesterol efflux in cultured macrophages and similarly impaired the process in vivo both when mice were injected with apoE expressing (MPM)- and not expressing (J774)-macrophages.

In conclusion, this study provided the evidence that CsA inhibits the antiatherosclerotic process of macrophage RCT in vivo. We propose that this effect is associated with the inhibition of CYP7Α-mediated elimination of cholesterol from the body. The current work suggests that the increase of cardiovascular risk in CsA-treated subjects may be associated, at least in part, with the impairment of macrophage RCT.

## Supporting Information

Figure S1 CsA treatment effect on plasma lipid levels in mice receiving J774. C57BL/6 mice were treated with CsA 50 mg/kg/d (black bar) or vehicle (white bar) for 14 days. The day before the sacrifice, mice were intraperitoneally injected with [^3^H]-cholesterol-loaded J774, in order to quantify macrophage RCT in vivo. Total cholesterol (TC), HDL-cholesterol (HDL-C) and triglycerides (TG) were measured by an enzymatic assay on plasma samples, as described in the Methods section. Non HDL- cholesterol (non HDL-C) is calculated as the difference between TC and HDL-C. Data are expressed as mean ± SD (values are mean of 5 animals).
(TIF)

Figure S2 CsA effect on cholesterol efflux from Caco-2 cells. Caco-2 cells were cultured on membranes transwell plates for 2 weeks. After the differentiation period, cells were labeled with [^3^H]-cholesterol 2 µCi/ml for 24 h and successively equilibrated in presence or absence of T0901317 10 µM for 24 h. A: Cholesterol efflux was promoted to cell medium for 24 h. CsA 5 µM was added during the equilibration (eq.) or the efflux (eff.) period. B: Cholesterol efflux was promoted in presence or absence of taurocholate (TA)-containing micelles (5 mM) and CsA (5 µM), for 24 h. [^3^H]-cholesterol released into the apical medium was measured by liquid scintillation counting. Efflux is expressed as cpm in medium/cpm time zero x100 (mean of triplicates ± SD). A: ## p<0.01 vs untreated cells; *p<0.05 vs T0901317-treated cells. B: *p<0.05, **p<0.01, *** p<0.0001 vs untreated cells; #p<0.05, ## p<0.01 vs T0901317-treated cells.
(TIF)

Table S1 Effect of 14 day treatment with CsA on body weight in mice injected with MPM. C57BL/6 mice were treated with CsA as described in Figure 1. Body weight was measured at baseline, on day 7 and on day 14 of the pharmacological treatment. Data are presented as mean ± SD (n = 7).
(DOCX)

Table S2 Effect of 14 day treatment with CsA on liver weight in mice injected with MPM. C57BL/6 mice were treated with CsA as described in Figure 1. On day 14 of the pharmacological treatment, mice were sacrificed and liver was collected after perfusion with a saline solution. Data are presented as mean ± SD of the wet weight. (n = 7).
(DOCX)

Table S3 Effect of 14 day treatment with CsA on body weight in mice injected with J774. C57BL/6 mice were treated with CsA as described in Figure 1. Body weight was measured at baseline, on day 7 and on day 14 of the pharmacological treatment. Data are presented as mean ± SD (n = 5).
(DOCX)

Table S4 Effect of 14 day treatment with CsA on liver weight in mice injected with J774. C57BL/6 mice were treated with CsA as described in Figure 1. Body weight was measured at baseline, on day 7 and on day 14 of the pharmacological treatment. Data are presented as mean ± SD (n = 5).
(DOCX)

File S1 (DOCX)
