# Supplementary figures and images for: Cyclosporine A Impairs the Macrophage Reverse Cholesterol Transport in Mice by Reducing Sterol Fecal Excretion
Source: PLoS One. 2013 Aug 9;8(8):e71572. doi: 10.1371/journal.pone.0071572 (PMC3739729; doi:10.1371/journal.pone.0071572)

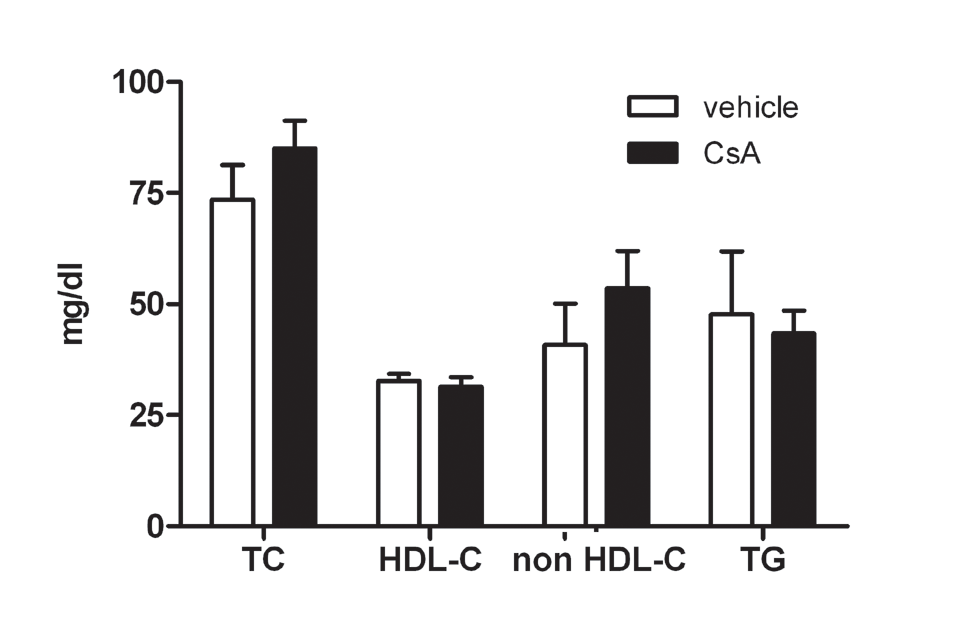

Supplement: Figure S1 — CsA treatment effect on plasma lipid levels in mice receiving J774. C57BL/6 mice were treated with CsA 50 mg/kg/d (black bar) or vehicle (white bar) for 14 days. The day before the sacrifice, mice were intraperitoneally injected with [3H]-cholesterol-loaded J774, in order to quantify macrophage RCT in vivo. Total cholesterol (TC), HDL-cholesterol (HDL-C) and triglycerides (TG) were measured by an enzymatic assay on plasma samples, as described in the Methods section. Non HDL- cholesterol (non HDL-C) is calculated as the difference between TC and HDL-C. Data are expressed as mean ± SD (values are mean of 5 animals). (TIF) [file pone.0071572.s001.tif]
