# Supplementary material for: Cyclosporine A Impairs the Macrophage Reverse Cholesterol Transport in Mice by Reducing Sterol Fecal Excretion
Source: PLoS One. 2013 Aug 9;8(8):e71572. doi: 10.1371/journal.pone.0071572 (PMC3739729; doi:10.1371/journal.pone.0071572)
Supplement: Table S1 — Effect of 14 day treatment with CsA on body weight in mice injected with MPM. C57BL/6 mice were treated with CsA as described in Figure 1. Body weight was measured at baseline, on day 7 and on day 14 of the pharmacological treatment. Data are presented as mean ± SD (n = 7). (DOCX) [file pone.0071572.s003.docx]

**Table S1: effect of 14 day treatment with CsA on body weight in mice injected with MPM**

|  | **Day 0 (g)** | **Day 7 (g)** | **Day 14 (g)** |
| --- | --- | --- | --- |
| **Vehicle** | 26.2±2.1 | 25.3±1.1 | 24.5±1.0 |
| **CsA** | 26.0±2.1 | 25.4±2.7 | 25.3±2.0 |

CsA: Cyclosporine A
