# Supplementary material for: Cyclosporine A Impairs the Macrophage Reverse Cholesterol Transport in Mice by Reducing Sterol Fecal Excretion
Source: PLoS One. 2013 Aug 9;8(8):e71572. doi: 10.1371/journal.pone.0071572 (PMC3739729; doi:10.1371/journal.pone.0071572)
Supplement: Table S2 — Effect of 14 day treatment with CsA on liver weight in mice injected with MPM. C57BL/6 mice were treated with CsA as described in Figure 1. On day 14 of the pharmacological treatment, mice were sacrificed and liver was collected after perfusion with a saline solution. Data are presented as mean ± SD of the wet weight. (n = 7). (DOCX) [file pone.0071572.s004.docx]

**Table S2: effect of 14 day treatment with CsA on liver weight in mice injected with MPM**

|  | **Weight (g)** | **Mean±S.D.** |
| --- | --- | --- |
| **Vehicle 1** | 0.976 | 1.089±0.138 |
| **Vehicle 2** | 1.229 |  |
| **Vehicle 3** | 1.249 |  |
| **Vehicle 4** | 1.069 |  |
| **Vehicle 5** | 1.123 |  |
| **Vehicle 6** | 0.856 |  |
| **Vehicle 7** | 1.119 |  |
| **CsA 1** | 1.217 | 1.158±0.093 |
| **CsA 2** | 1.318 |  |
| **CsA 3** | 1.145 |  |
| **CsA 4** | 1.101 |  |
| **CsA 5** | 1.186 |  |
| **CsA 6** | 1.033 |  |
| **CsA 7** | 1.108 |  |

CsA: Cyclosporine A
