# Supplementary material for: Cyclosporine A Impairs the Macrophage Reverse Cholesterol Transport in Mice by Reducing Sterol Fecal Excretion
Source: PLoS One. 2013 Aug 9;8(8):e71572. doi: 10.1371/journal.pone.0071572 (PMC3739729; doi:10.1371/journal.pone.0071572)
Supplement: Table S3 — Effect of 14 day treatment with CsA on body weight in mice injected with J774. C57BL/6 mice were treated with CsA as described in Figure 1. Body weight was measured at baseline, on day 7 and on day 14 of the pharmacological treatment. Data are presented as mean ± SD (n = 5). (DOCX) [file pone.0071572.s005.docx]

**Table S3: effect of 14 day treatment with CsA on body weight in mice injected with J774**

|  | **Day 0 (g)** | **Day 7 (g)** | **Day 14 (g)** |
| --- | --- | --- | --- |
| **Vehicle** | 25.2±1.1 | 25.9±1.8 | 24.1±1.1 |
| **CsA** | 24.9±1.1 | 24.6±0.8 | 23.4±1.2 |

CsA: Cyclosporine A
