# Supplementary material for: Cyclosporine A Impairs the Macrophage Reverse Cholesterol Transport in Mice by Reducing Sterol Fecal Excretion
Source: PLoS One. 2013 Aug 9;8(8):e71572. doi: 10.1371/journal.pone.0071572 (PMC3739729; doi:10.1371/journal.pone.0071572)
Supplement: Table S4 — Effect of 14 day treatment with CsA on liver weight in mice injected with J774. C57BL/6 mice were treated with CsA as described in Figure 1. Body weight was measured at baseline, on day 7 and on day 14 of the pharmacological treatment. Data are presented as mean ± SD (n = 5). (DOCX) [file pone.0071572.s006.docx]

**Table S4: effect of 14 day treatment with CsA on liver weight in mice injected with J774**

|  | **Weight (g)** | **Mean±S.D.** |
| --- | --- | --- |
| **Vehicle 1** | 0.963 | 1.106±0.109 |
| **Vehicle 2** | 1.266 |  |
| **Vehicle 3** | 1.074 |  |
| **Vehicle 4** | 1.098 |  |
| **Vehicle 5** | 1.311 |  |
| **CsA 1** | 1.248 | 1.309±0.186 |
| **CsA 2** | 1.351 |  |
| **CsA 3** | 1.174 |  |
| **CsA 4** | 1.612 |  |
| **CsA 5** | 1.160 |  |

CsA: Cyclosporine A
