# Supplementary material for: Cyclosporine A Impairs the Macrophage Reverse Cholesterol Transport in Mice by Reducing Sterol Fecal Excretion
Source: PLoS One. 2013 Aug 9;8(8):e71572. doi: 10.1371/journal.pone.0071572 (PMC3739729; doi:10.1371/journal.pone.0071572)
Supplement: File S1 — (DOCX) [file pone.0071572.s007.docx]

**Supporting Information**

**Methods**

*Cells*

Human epithelial colorectal adenocarcinoma (Caco-2) cells, a validated cell model for lipid efflux experiments [1], were a kind donation from Dr. Franca Zani (Dipartimento di Farmacia, Università degli Studi di Parma).

*Cholesterol efflux from Caco-2 cells*

Caco-2 cells were cultured on polycarbonate semipermeable Transwell membranes (Greiner Bio-one, Frickenhausen, Germany) with 0.4 µm pores at a density of 2.0 x 10^5^ cells/well in 12-well culture membrane for 14 days in medium containing 10% fetal calf serum. Every 3 or 4 days, cell culture medium was renewed. After the differentiation period, cell monolayers were labeled with 2µCi/ml [^3^H]-cholesterol in medium in the presence of 10% fetal calf serum for 24h. *Abcg5/Abcg8* transporter expression was induced by incubation with the synthetic Liver X Receptor ligand T0901317 10 µM, during equilibration period of 24h. Efflux was promoted to culture medium in absence of extracellular acceptor or in presence of taurocholate micelles (5 mM), for 24h. In the first case, CsA 5µM was added during either the equilibration or the efflux period. In the second case, CsA was added only in the efflux period together with micelles. To prepare 5mM taurocholate micellar solutions, appropriate volumes of the stock solutions prepared in ethanol were evaporated under nitrogen and the dried lipids were dissolved in DMEM. The resulting solution was stirred vigorously at 37°C until clear [2]. Cholesterol efflux percentages were obtained by measuring the release of radiolabeled cholesterol into the medium, as described previously [3]. The medium from apical and basal chambers were collected and centrifuged at 15000 rpm for 10 min to obtain supernatants. The cells, washed three times with PBS, were harvested and lysed in 0.1% TritonX-100-PBS. Radioactivity secreted in the media and retained in the cells were quantified by liquid scintillation counting. The cholesterol efflux rate was calculated by dividing radioactivity in the apical medium in 24h over the radioactivity incorporated by cells (Time zero).

**References**

1. Field FJ, Watt K, Mathur SN (2010) TNF-alpha decreases ABCA1 expression and attenuates HDL cholesterol efflux in the human intestinal cell line Caco-2. J Lipid Res 51: 1407-1415.

2. Mathur SN, Watt KR, Field FJ (2007) Regulation of intestinal NPC1L1 expression by dietary fish oil and docosahexaenoic acid. J Lipid Res 48: 395-404.

3. Tachibana S, Hirano M, Hirata T, Matsuo M, Ikeda I, et al. (2007) Cholesterol and plant sterol efflux from cultured intestinal epithelial cells is mediated by ATP-binding cassette transporters. Biosci Biotechnol Biochem 71: 1886-1895.
